# Interlaboratory Comparative Tests in Ready-Mixed Concrete Quality Assessment

**DOI:** 10.3390/ma14133475

**Published:** 2021-06-22

**Authors:** Izabela Skrzypczak, Agnieszka Leśniak, Piotr Ochab, Monika Górka, Wanda Kokoszka, Anna Sikora

**Affiliations:** 1 Faculty of Civil and Environmental Engineering and Architecture, Rzeszow University of Technology, Powstancow Warszawy 12, 35-082 Rzeszow, Poland; izas@prz.edu.pl (I.S.); p.ochab@prz.edu.pl (P.O.); wandak@prz.edu.pl (W.K.); sikora@prz.edu.pl (A.S.); 2 Faculty of Civil Engineering, Cracow University of Technology, 31-155 Krakow, Poland; monika.gorka@pk.edu.pl

**Keywords:** ready-mixed concrete, construction architecture material, inter-laboratory comparisons (ILC), proficiency testing (PT), concrete quality assessment

## Abstract

Proper quality assessment of ready-mixed concrete, which is currently the principal material for construction, land engineering and architecture, has an impact on the optimisation and verification of correct functioning of individual stages of the production process. According to the European Standard EN 206 “Concrete–Specification, performance, production and conformity”, obligatory conformity control of concrete is carried out by the producer during its production. In order to verify the quality of concrete, investors generally commission independent laboratory units to perform quality assessment of both concrete mix and hardened concrete, which guarantees a high quality of construction works. One of the essential tools for ensuring the quality of test results is the participation of laboratories in the so-called proficiency testing (PT) or inter-laboratory comparisons (ILC). Participation in PT/ILC programmes is, on the one hand, a tool for demonstrating the laboratory’s performance, on the other hand an aid for maintaining the quality of available concrete tests and validating test methods. Positive evaluation is a confirmation of the laboratory’s capability for performing the tests. The paper presents the results of laboratory proficiency tests carried out by means of inter-laboratory comparisons, as shown in the example of quality assessment of ready-mixed concrete for nine participating laboratories. The tests were performed for concrete of the following parameters: strength class C30/37, consistency S3, frost resistance degree F150, and water resistance degree W8. This involved determining consistencies, air content and density of the concrete mix, and compressive strength of hardened concrete. For the evaluation of laboratory performance results, *z-score*, *ζ-score* and *E_n_-score* were applied. The innovation of the proposed study lies in employing both classical and iterative robust statistical methods. In comparison with classical statistical methods, robust methods ensure a smaller impact of outliers and other anomalies on the measurement results. Following the analyses, clear differences were found between the types of detected discrepancy of test results, which occurred due to the nature of individual parameters. For two laboratories, two scores revealed unsatisfactory results for concrete mix consistency. The main reasons can be pouring into the cone-shaped form a concrete mixture that is too dry, or incorrect use of a measuring tool also creating a possibility that the obtained value can be wrongly recorded. Other possible reasons are discussed in the paper. Participation in inter-laboratory comparison programmes is undoubtedly a way to verify and raise the quality of tests performed for concrete mix and hardened concrete, whereas individual analysis of the results allows the laboratory quality system to be improved.

## 1. Introduction

Modern structures must meet various design standards concerning durability, ergonomics, safety and quality; furthermore, construction has to be executed without harm to the environment and natural resources. With the development of technology and advanced construction materials, increasingly more requirements are being imposed. Modern construction consists of structures of possibly minimal impact on the environment [1,2,3], which is achieved through proper architectural design, use of proper construction materials characterised, among other things, by low CO_2_ emission [4,5,6], low energy consumption during operation and construction [7,8,9], and the possibility of renovation following extensive use [10,11] or potential malfunction [12,13]. Not without significance is also the use of construction materials that can be recycled or utilised [14,15]. Hence, both the design and execution of modern structures requires undertaking measures that involve employing appropriate quality assessment processes. Assessment is conducted with regard to both the production of construction materials, and the process of design, execution and completion of construction works or entire structures. Each of these procedures is of different character. Quality control of construction materials delivered to the market is limited by legal provisions, whereas the remaining measures related to the execution of construction objects can be approached optionally while remaining within the framework of internal, domestic procedures [16]. Concrete is undoubtedly one of the most frequently used construction materials [17]. According to a Chatham House report [18], the world produces 4.4 billion tons of concrete annually, but that amount is expected to rise to over 5.5 billion tons by 2050. Ready-mixed concrete (RMC) is the principal construction material for civil engineering infrastructure [19]. It should be noted that construction concrete that is subject to quality control constitutes circa 70% of total concrete production [20]. Quality assessment may be carried out on various stages of production, delivery, and before and after laying concrete mix. The intended concrete quality is achieved owing to the selection of appropriate formulas (ingredients, strength class, exposure class etc.) [21,22,23,24,25,26], a production process that is compliant with the procedures [27,28,29], the mode of transport and laying of fresh concrete mix, and the proper maintenance of hardened concrete [30,31]. Quality assessment involves the control of conformity and uniformity in compliance with the recommended criteria. Not without significance for the quality of modern concretes remains the development of innovative research methods that aid concrete design aimed at obtaining appropriate properties and durability [32,33,34,35], and the development of methods for the evaluation of the results [16,36].

Concrete testing procedures and quality assurance criteria for ready-mixed concrete delivered to the construction site are fairly well-established under law, especially under industry standards. It is also important to underscore the significance and role of technical specification for execution and completion of construction works. Although appropriate industry standards contain conformity and uniformity criteria related to concrete production control, technical specifications contain, in particular, sets of requirements that are necessary for determining the standard and quality of works with regard to the manner of execution of construction works and the evaluation of correctness of execution of individual stages; these elaborations are custom-developed for each construction project [37].

Industry standards require that the concrete supplier implement a production and quality control plan—a set of quality requirements with regard to the products and their production. The plan contains a detailed description of the manufacturing process which includes stages, organisation, methods and standards of production as well as procedures and instructions, and the testing and quality control programme [38,39,40,41,42,43]. For the ready-mixed concrete supplier, quality assessment involves pro-active measures that allow for maintaining the quality of concrete by ensuring the cohesion of properties of concrete mix and hardened concrete between the batches and for the entire duration of the project; quality assessment also includes appropriate actions and measures taken in the case when the supplied product does not meet the requirements [38]. In construction practice, in the execution of concrete works, concrete quality is usually verified by the investor by commissioning an independent laboratory unit to carry out the tests, which is a guarantee for quality control of both concrete mix and hardened concrete. As set out by ISO/IEC 17000:2020, “Conformity assessment–Vocabulary and general principles” [44], accreditation is “attestation by a third party, related to the unit assessing conformity, providing formal evidence of its competence for executing defined tasks within the scope of conformity assessment”. The IOS 17000:2020 standard contains a requirement that the laboratories have quality control procedures and plan their actions, which would subsequently be subject to monitoring. The actions should ensure the reliability of test results delivered to the customer. One of the essential tools for ensuring the quality of test results is the laboratories’ participation in proficiency testing (PT) or inter-laboratory comparison (ILC) programmes [45]. In accordance with EN ISO/IEC 17025 [46] and EA-4/18 [47], accredited laboratories should ensure the quality of the results through participation in proficiency testing programmes. Involvement in PT/ILC programmes is, on the one hand, a tool for demonstrating the laboratory’s performance, and on the other hand an aid for maintaining the quality of available tests and validating test methods. Participation in comparative PT/ILC programmes is usually paid for, and the services are provided by domestic and international organisers of PT/ILC programmes. Laboratories may, however, organise inter-laboratory comparisons with other laboratories on their own behalf. Such activity is not aimed at qualifying or evaluating the operation of participating laboratories, yet it allows the obtained results to be analysed individually and used to improve testing quality. In practice, inter-laboratory testing is most frequently organised for one of the three following purposes [48,49,50]: assessment of laboratory proficiency, certification of reference material, evaluation (validation) of analysis method. Participation in inter-laboratory comparisons undoubtedly contributes to improving quality systems in laboratories. This directly translates into the quality of assessment, for instance of concrete, in the monitored facilities.

The aim of the present paper is the evaluation of laboratory performance by means of inter-laboratory proficiency tests as carried out for ready-mixed concrete quality assessment, for nine participating laboratory units. The most innovative element of the study was the simultaneous use of classical and robust statistical methods. In practice, the most common procedures employ classical statistical analysis, which is optimal under the assumption of normality of data distribution and large sample size. Classical statistical procedures involve, as a first step, the verification of questionable results, e.g., by applying the Grubbs test, which allows us to identify and remove abnormal values, i.e., outliers. This is only effective for large data sets, whereas for small-sized samples, removing one or several significant outliers may greatly alter the classical statistical parameters. In many fields of experimental research, particularly in destructive testing, the tests are limited to small-sized samples due to high cost intensity of the testing process. In such circumstances, it is necessary to make use of all the obtained measurement results, as removing outliers/questionable results from the sample diminishes the reliability of statistical assessment. Employing robust statistical methods is recommended, as, in comparison with classical statistical methods, they ensure a smaller impact of outliers and other anomalies on the measurement results. Quality assessment of concrete is generally carried out on the basis of small-sized samples, by means of destructive testing, without the possibility to repeat or complement the measurements. This is why iterative robust statistical methods, rarely used in concrete quality control, were proposed for the analysis of inter-laboratory comparative tests. Southern Poland-based laboratories participated in the programme voluntarily. Concrete tests and analyses were performed from June to July 2020.

## 2. Materials and Methods

### 2.1. Laboratory Proficiency Testing/Interlaboratory Comparison

**Figure 1:**
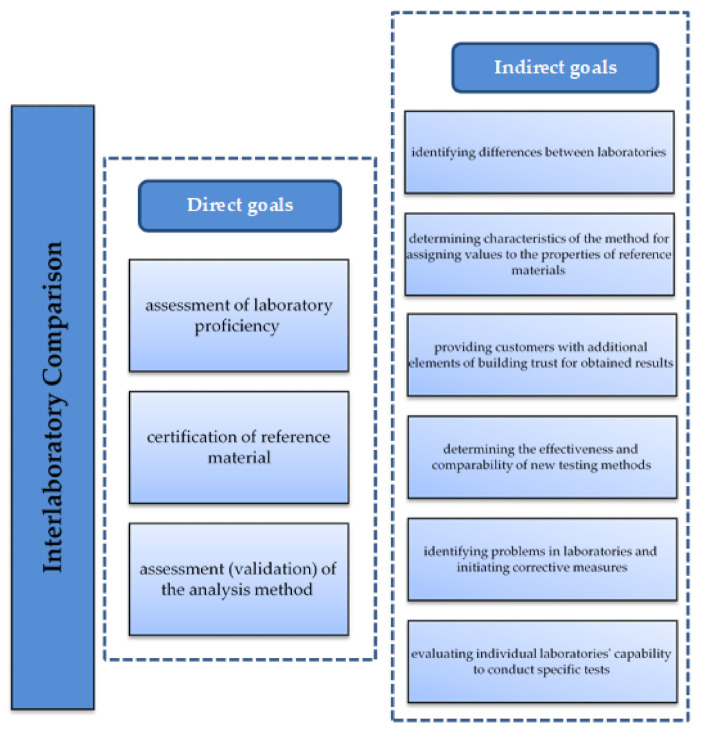
Goals of inter-laboratory comparisons. Source: own elaboration based on [53,54].

According to ISO/IEC 17043:2010 [51], laboratory proficiency testing involves the evaluation of a participant’s performance against pre-established criteria by means of inter-laboratory comparisons. This allows the laboratory’s capability for conducting concrete tests to be assessed, and thus the reliability of the obtained results to be evaluated. In proficiency testing, the results of the analyses of the same object, obtained in a given laboratory, are compared with the results obtained independently in one or several different laboratories. The basic tool for carrying out proficiency testing is inter-laboratory comparison, which involves the organised conduct and evaluation of testing of the same or similar objects by at least two laboratories, in accordance with previously determined conditions [52]. Furthermore, inter-laboratory comparisons can be used for different, indirect purposes, as shown in Figure 1.

Participation in proficiency testing programmes constitutes, therefore, an evaluation of a laboratory’s testing capabilities. It provides an independent, external assessment, which complements the laboratory’s internal quality control procedures. Participation in a proficiency testing programme may be a basis for self-evaluation, and contribute to improving the laboratory’s proficiency; it is, thus, an important, often obligatory element of the laboratory’s quality control system.

Prior to proceeding with the testing, an appropriately designed and organised programme must be prepared in order to determine the type of analytical methods, the type of test object, and the number of participating laboratories.

While preparing the test object, one should take into consideration all factors that may influence the reliability of inter-laboratory testing, such as the object’s homogeneity, the method of sample collection, the object’s stability over time, and the impact of environmental conditions (during transport and storage) on the test object’s properties. It is crucial that the material used in proficiency testing is homogenous, and that all participants are provided with test objects which do not significantly differ in terms of tested parameters. The material’s uniformity should be documented, and the conditions of its transportation and storage, and the timeframe of the testing, should be clearly defined. Test methods should be unambiguously determined and based on normalised and validated methods.

### 2.2. Evaluation of Comparative Test Results

The results obtained by the participants are subject to statistical analysis and evaluation. The methods for statistical evaluation of inter-laboratory comparison results are described in the following standards:

- ISO/IEC 17043 (2010) “Conformity assessment—general requirements for proficiency testing” [51];
- ISO 13528 (2015) “Statistical methods for use in proficiency testing by inter-laboratory comparison” [55].

Most of the methods are based on a known assigned value (value attributed to a particular quantity and accepted [55] and its uncertainty. Assigned value (*x_pt_*) is the value agreed on the basis of the participants’ results in the way described in ISO/IEC 17043 (2010) [51], Annex B.2.1 e) and ISO 13528:2015 [55], Annex C, Algorithm A p.C.3.1.

Assigned value is calculated as arithmetic mean from the participants’ results, having considered the influences of outliers, with the use of robust statistical methods.

Standard uncertainty of assigned value *u*(*x_pt_*) is determined with the application of statistical method as described in ISO 13528: 2015 p.7.7.3 [55] and calculated by Equation (1):

(1) $$u\left(x_{pt}\right)=1.25\cdot \frac{S^{*}}{\sqrt{p}}$$

where: *p*—number of participants,

S*—strong (solid) standard deviation calculated by Equation (2):

(2) $$S^{*}=1.134\;\sqrt{\frac{\sum {\left(x_{i}^{*}-x_{pt}\right)}^{2}\;}{p-1}}$$

where: x_i_*—results obtained by the participants after applying robust statistics,

*x_pt_*—assigned value, calculated as strong (solid) mean from the participants’ results.

Expanded uncertainty (*U_r_*) of the assigned value, with expansion coefficient k = 2 and confidence level circa 95%, is calculated by Equation (3):

(3) $$U_{r}=2\cdot U\left(x_{pt}\right)$$

### 2.3. Means of Proficiency Assessment and Evaluation Criteria for Laboratory Activity Results

Laboratory activity results are usually presented with the use of *z-score, ζ-score* and *E_n_-score,* which are determined in accordance with ISO/IEC 17043: 2011 [51], Annex B, B.3.1.3 c) and d), and calculated by Equations (4)–(6):

- *z-score* (4):
  (4) $$z=\frac{x_{i\;}-x^{*}}{SD}$$

where: *SD*—standard deviation for proficiency test assessment, determined considering the results of all participants,

*x_i_*—result reported by the participant,

x*—assigned value, determined as strong (robust) mean from the participants’ results.

- *ζ-score* (5):
  (5) $$\zeta =\frac{x_{i\;}-x^{*}}{\sqrt{\mu_{x_{i}}{}^{2}+\mu_{x^{*}}{}^{2}\;}}$$

where: $x_{i}$—result reported by the participant,

$x^{*}$—assigned value, determined as strong (robust) mean from the participants’ results,

$\mu_{x_{i}}$—standard uncertainty estimated by the participant,

$\mu_{x^{*}}$—standard uncertainty of assigned value $x^{*}$.

- *E_n_-score* (6):
  (6) $$E_{n}=\frac{x_{i\;}-x^{*}}{\sqrt{U_{x_{i}}{}^{2}+U_{x^{*}}{}^{2}\;}\;}\;$$

where: $x_{i\;}$—result reported by the participant,

$x^{*}$—assigned value, determined as strong (solid) mean from the participants’ results,

$U_{x_{i}}$—measurement uncertainty estimated by the participant,

$U_{x^{*}}$—measurement uncertainty of assigned value $x^{*}$.

The results of the laboratories’ activity are evaluated with the use of *z-score*. By assessing a participant’s performance by means of *z-score*, both the trueness and precision of the obtained result are addressed [52]. *ζ-score* and *E_n_-score* can be applied in combination with *z-score* as an aid for improving the laboratories’ activity [56,57]. *ζ-score and E_n_-score* depend on the participants submitting accurate measurement uncertainty estimates along with their result, a procedure not easily adhered to [52].

Assessment according to *z-score*, *ζ-score* and *E_n_-score* is applied to all results, including those which, as outliers, were not considered in statistical calculations of the assigned value and its standard deviation. The results of actions of the programme’s participants were evaluated according to the following criteria (Table 1):

Is it worth mentioning that the paper [45] presented an approach toward the analysis of inter-laboratory comparison results for a small number of laboratories (2) and small number of samples (3), which can apply for e.g., a construction product for which tests and test elements are very expensive.

### 2.4. Laboratory Proficiency Tests with Regard to Testing Concrete Mix and Hardened Concrete

The tests involved the participation of nine southern Poland-based laboratory units. For confidentiality reasons, the present elaboration did not include the names or addresses of the laboratories. The proficiency testing programme considered test objects, measured parameters, and methods of testing concrete mix and hardened concrete as shown in Table 2.

The laboratory proficiency testing programme was designed following the guidelines set out by ISO 13528:2015 [55], Annex B, and consisted in the collection of samples of concrete mix, and the preparation and maintenance of concrete samples by each of the participating laboratories. This is why the concrete mix was the only area of uniformity and stability control of the test object. For concrete sample testing, together with sample collection, inter-laboratory assessment involves the preparation and maintenance of concrete samples, and all related measures, including the transport of samples to the participants’ laboratories. For concrete samples, the test object’s instability effects were eliminated, as the testing programme assumed that the preparation, maintenance and assessment of concrete samples should be carried out by each participant at the same time (concrete compressive strength test—28 days after preparing test forms).

As the properties of concrete mix change over time, the quickest possible method was adopted for collecting samples. It was assumed that all samples would be collected within circa 15 min, in a single place.

Due to the availability, universality and cost-efficacy of testing methods, immediate tests of consistency and/or air content were employed to determine uniformity and stability of concrete mixes. For all participants, the manner of sample collection, preparation, testing and transportation complied with the recommendations set out by the relevant industry standards (Table 1) and programme-specific guidelines. Comparative tests were performed for concrete of the following parameters: strength class C30/37, consistency S3, frost resistance degree F150 and water resistance degree W8.

## 3. Results and Discussion

**Figure 2:**
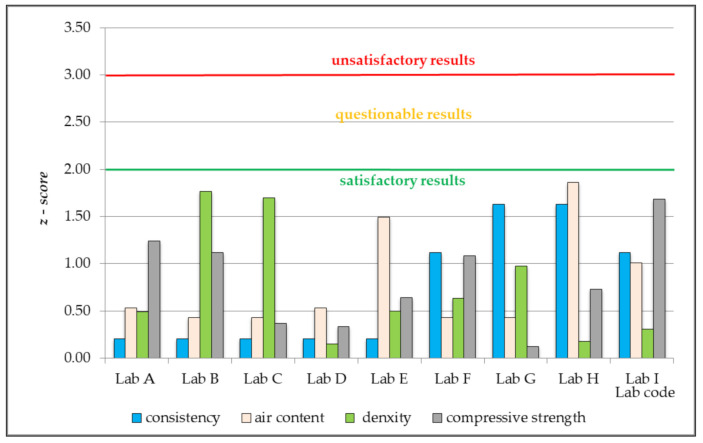
The value of z-score based on the results of the tests conducted by the participating laboratories, which performed measurements of density of concrete mix and compressive strength of hardened concrete according to the classical statistical method.

**Figure 3:**
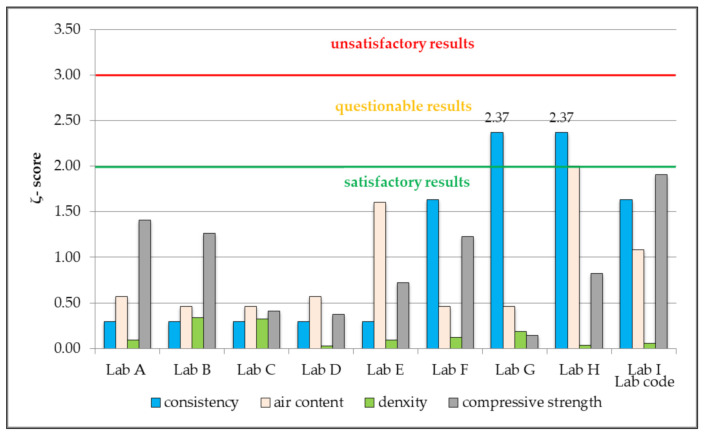
The value of *ζ-score* based on the results of the tests conducted by the participating laboratories, which performed measurements of density of concrete mix and compressive strength of hardened concrete according to the classical statistical method.

**Figure 4:**
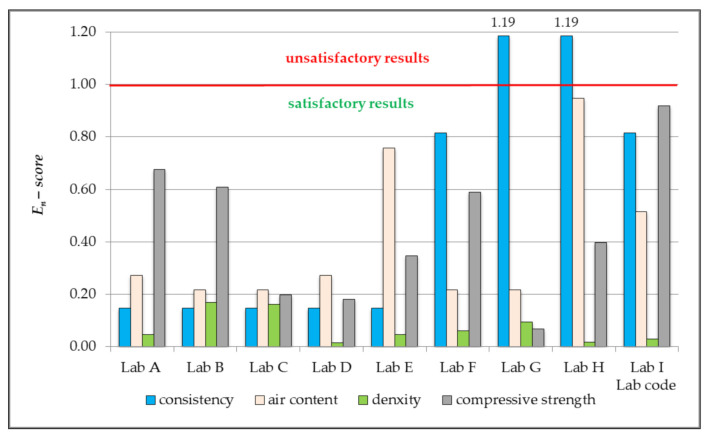
The value of *E_n_*-score based on the results of the tests conducted by the participating laboratories, which performed measurements of density of concrete mix and compressive strength of hardened concrete according to the classical statistical method.

Based on the test results obtained by each of the participating laboratories, a number of parameters was calculated for the purpose of statistical quality assessment of performed measurements, in compliance with the recommendations of individual industry standards [38,39,40,41,42,43] and the testing programme designed according to [53,55]. The most important parameters for laboratory assessment are: standard deviation, expanded uncertainty, and *z-score*, ζ-*score* and *E_n_-score*. Calculated values were presented in Table 3, Table 4, Table 5, Table 6 and Table 7 and Figure 2, Figure 3 and Figure 4.

In data analysis and inter-laboratory comparative testing, various statistical tests, e.g., the Grubbs test, are performed to identify and remove outliers. This is appropriate and effective only for large samples. For concrete mix and hardened concrete tests (small samples), removing some data items (from one to three test results) significantly decreases the accuracy of uncertainty estimation, which is why the conducted analyses employed robust statistical methods, designed for robustness against slight deviation from the model (particularly the occurrence of outliers) [56,57]. The calculations were performed in compliance with ISO 13528: 2009-01 “Statistical method for use in proficiency testing by inter-laboratory comparisons”, Annex C (normative) Robust Analysis [55]. The values of statistical parameters determined with the use of classical and robust methods were presented in Table 7.

The next step of the calculations was to determine, based on the obtained statistical parameters (Table 6), the values of individual measures that would allow the laboratories’ proficiency to be assessed, i.e., the values of *z-score, ζ-score* and *E_n_-score*. On the basis of the inter-laboratory proficiency tests performed, all participating southern Poland-based laboratories which carry out tests of concrete mix and hardened concrete conduct them on a satisfactory level, as confirmed by the obtained *z-score* values |*z*| < 2.0 (Figure 2).

The obtained values of *ζ-score* and *E_n_-score* for seven out of nine laboratories indicate satisfactory proficiency in determining consistency, air content and density of concrete mix, and compressive strength of hardened concrete (Figure 3 and Figure 4).

Only for two laboratories, defined as Lab G and Lab H, the obtained values of *ζ-score* and *E_n_-score* reveal unsatisfactory application by the participants of the methods for testing and consistency determination. The value of *ζ-score* indicates that the results obtained for the consistency parameter are questionable, whereas the value of *E_n_-score* shows the results to be unsatisfactory (Figure 3 and Figure 4).

In both cases, the limit value for *ζ-score* was exceeded by 18.5% → *ζ score* = 2.37, while the limit value for *E_n_-score* was exceeded by 19% →*E_n_-score* = 1.19. This is not a significant exceeding of a satisfactory value, but one that constitutes a questionable result, which suggests undertaking appropriate measures with the aim to establish the causes of incorrect assessment of the consistency of concrete mix. Questionable results might be, in this case, caused by pouring into the cone-shaped form a concrete mixture that is too dry, or incorrect use of a measuring tool. Most tests, such as the slump flow test and the slump test, are carried out by operators by using a ruler or a stopwatch, which is why measurement errors are inherent, and the measured value may vary depending on the operator [58]. There is also a possibility that the obtained value can be wrongly recorded or easily manipulated after measurement. If concrete of insufficient workability is used for construction due to inaccurate measurement results or incorrect data records, it will cause future problems in terms of structural safety [58,59]. Consistency determines the ease of mixing concrete in a form for a given method of laying concrete. Fluidity parameters should comply with the overall plan of the project, as proper concrete consistency allows the durability of a structure to be predicted. Monitoring concrete fluidity [60,61] and durability [62,63,64] is considered crucial in long-distance transport of concrete required for constructing tower blocks and long-span bridges.

**Figure 5:**
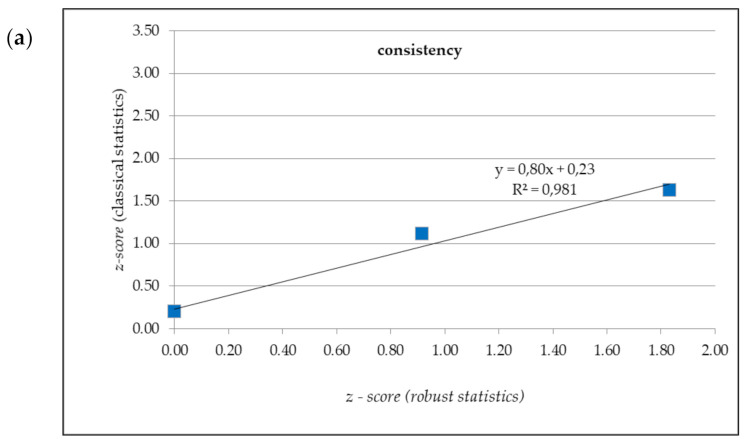

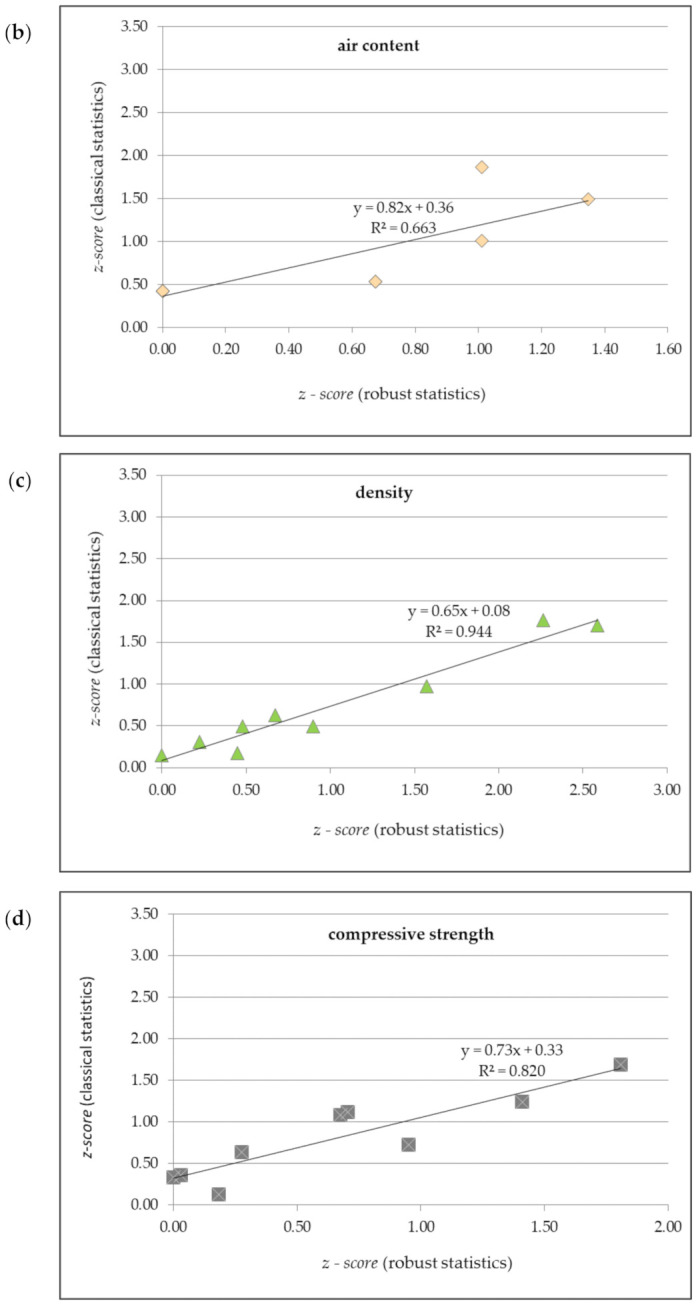
Regression functions of *z-score* in classical statistics and *z-score* in robust statistics for various parameters: (**a**) consistency; (**b**) air content; (**c**) density; (**d**) compressive strength.

For *z-score* values determined with the use of classical and robust statistical method, regression and correlation analysis was performed. Figure 5 shows graphs of regression functions of *z-score* in classical statistics and *z-score* in robust statistics for various parameters.

High positive correlation can be observed between *z-score* in classical statistics and *z-score* in robust statistics. Pearson product–moment correlation coefficients for *z-score* in classical statistics versus z-score in robust statistics are presented in Table 8.

Combination of classical and robust statistical methods with the use of *z-score* can reduce the risks related to laboratory activities. In classical and robust statistics, *z-score* parameters, based on an assigned value, are more effective in detecting a laboratory having outlier results. *Z-score*, which is based on the difference between the reported result and the assigned value, is particularly useful for detecting discrepancies between laboratories, and may prove helpful in improving their activities. Neither of these methods nor their combination guarantee proper assessment, and they should not be used for the main assessment of laboratory performance in inter-laboratory comparisons. Methods for robust estimation in small samples do not improve the efficiency of the *z-score* parameter in detecting discrepancies of test results. In ISO 13528 [55], Annex D1, it is underscored that some of the procedures for performance evaluation are unreliable when used for too small a number of participants. The conclusions are consistent with the information given in ISO 13528 [55] and in [45]. Assessment of reliability of small sample size tests is a difficult problem to solve in inter-laboratory comparisons of ready-mixed concrete. In such circumstances, it seems justified to refrain from activities aimed at ensuring testing quality by means of inter-laboratory comparisons, and focus on other aspects, such as the personnel’s competencies and equipment suitability. However, laboratories, particularly those responsible for carrying out tests of ready mix concrete that affect construction safety and quality, tend to be concerned about the correctness of test results. An inter-laboratory comparison could help them assess whether differences between laboratories are significant, and gain more confidence in their results. Such comparisons—as presented in the paper—give both the laboratory and its customer a slightly higher sense of security.

## 4. Conclusions

Proper quality control of concrete does not only influence the optimisation and verification of correct functioning of individual stages of the production process, but also directly impacts certification related to factory production control. Quality control is the basis for ensuring that the production plant meets at least the requirements and recommendations set out by EN 206:2014 “Concrete–Specification, performance, production and conformity”. The standard introduced a novel approach toward designing the composition and planning the production of concrete mixes, and evaluating concrete with regard to technical parameters. In turn, quality control through laboratory proficiency tests, for instance with regard to testing concrete mix and hardened concrete, allows individual laboratory units’ capability to perform specific research to be evaluated. For accredited laboratories, in order to monitor the reliability of the obtained results, participation in inter-laboratory comparison programmes or proficiency testing programmes is required by ISO/IEC 17025:2005. For the remaining research units, participation in such programmes is undoubtedly a means of verifying and improving the quality of conducted analyses, as well as a platform for exchanging experiences and views (after performing and documenting measurements), both for individual employees and the entire laboratory.

Comparative tests discussed in the present paper, conducted for nine participating laboratories, provided interesting data for scientific consideration. For the purpose of the tests, selected parameters/properties of concrete mix and hardened concrete were analysed. *Z-score* was applied to evaluate laboratory proficiency, whereas *ζ-score* and *E_n_-score* were used for improving the laboratories’ performance. For the laboratories defined as Lab G and Lab H, the results obtained provided information about irregularities, which made it possible to take adequate corrective measures with the aim to determine the causes of inappropriate assessment of concrete mix consistency.

Experiences gained from the conducted tests reveal a need to continue this type of project, with the cooperation of both past participants and new laboratory facilities, for the purpose of improving the quality of activities conducted by research units. A tangible aspect of regular participation in comparative tests, with positive results, is an increase in customer trust and the evidence of the laboratory personnel’s competencies.

## Figures and Tables

**Table 1:** Assessment of results according to the values of individual scores [52].

z-Score *	*ζ-Score*	*E_n_-Score*
$\left|z\right|$ ≤ 2.0–satisfactory result 2.0 < $\left|z\right|$ < 3.0–questionable result $\left|z\right|$ ≥ 3.0–unsatisfactory result	$\left|\zeta \right|$ ≤ 2.0–satisfactory result 2.0 < $\left|\zeta \right|$ < 3.0–questionable result $\left|\zeta \right|$ ≥ 3.0–unsatisfactory result	$\left|E_{n}\right|$ ≤ 1.0–satisfactory result $\left|E_{n}\right|$ > 1.0–unsatisfactory result

* Assessment according to z-score is not performed when the number of results for the tested parameter is lower than 8.

**Table 2:** Test objects, measured parameters and recommended standard test methods.

Proficiency Test Object	Measured Parameters/Properties	Test Method According to:
Concrete mix	Sample collection	EN 12350-1 [37]
	Consistency	EN 12350-2 [38]
	Concrete mix density	EN 12350-6 [40]
Concrete	Air content	EN 12350-7 [41]
	Compressive strength	EN 12390-3 [39]

**Table 3:** Table of obtained values of basic statistical parameters for individual participating laboratories with regard to measurements performed for concrete mix consistency with the use of classical statistical method.

Lab Code	Consistency Test Result mm	Mean Value mm	Standard Deviation mm	Expanded Uncertainty mm	Result Expressed as an Interval, as Reported by the Laboratory (Result Minus/Plus Measurement Uncertainty) mm
Lab A	130	128	11	15	115–145
Lab B	130				115–145
Lab C	130				115–145
Lab D	130				115–145
Lab E	130				115–145
Lab F	140				125–155
Lab G	110				95–125
Lab H	110				95–125
Lab I	140				125–155

**Table 4:** Table of obtained values of basic statistical parameters for individual participating laboratories with regard to measurements performed for concrete mix air content with the use of classical statistical method.

Lab Code	Air Content Test Result %	Mean Value %	Standard Deviation %	Expanded Uncertainty %	Result Expressed as an Interval, as Reported by the Laboratory (Result Minus/Plus Measurement Uncertainty) %
Lab A	1.6	1.7	0.2	0.3	1.3–1.9
Lab B	1,8				1.5–2.1
Lab C	1.8				1.5–2.1
Lab D	1.6				1.3–1.9
Lab E	1.4				1.1–1.7
Lab F	1.8				1.5–2.1
Lab G	1.8				1.5–2.1
Lab H	2.1				1.8–2.4
Lab I	1.5				1.2–1.8

**Table 5:** Table of obtained values of basic statistical parameters for individual participating laboratories with regard to measurements performed for concrete mix density with the use of classical statistical method.

Lab Code	Density Test Result kg/m^3^	Mean Value kg/m^3^	Standard Deviation kg/m^3^	Expanded Uncertainty kg/m^3^	Result Expressed as an Interval, as Reported by the Laboratory (Result Minus/Plus Measurement Uncertainty) kg/m^3^
Lab A	2287	2290	6	65	2222–2352
Lab B	2279				2214–2344
Lab C	2301				2236–2366
Lab D	2289				2224–2354
Lab E	2293				2228–2358
Lab F	2286				2221–2351
Lab G	2296				2231–2361
Lab H	2291				2226–2356
Lab I	2288				2223–2353

**Table 6:** Table of obtained values of basic statistical parameters for individual participating laboratories with regard to measurements performed for concrete compressive strength with the use of classical statistical method.

Lab Code	Compressive Strength Test Result MPa	Mean Value MPa	Standard Deviation MPa	Expanded Uncertainty MPa	Result Expressed as an Interval, as Reported by the Laboratory (Result Minus/Plus Measurement Uncertainty) MPa
Lab A	49.40	47.58	1.46	2.20	47.20–51.60
Lab B	45.95				43.75–48.15
Lab C	47.05				44.85–49.25
Lab D	47.10				44.90–49.30
Lab E	46.65				44.45–48.85
Lab F	46.00				43.80–48.20
Lab G	47.40				45.20–49.60
Lab H	48.70				46.50–50.90
Lab I	50.10				47.90–52.30

**Table 7:** Values of statistical parameters for individual parameters/properties of concrete mix and hardened concrete according to classical and robust statistics.

	Parameter/Property	Concrete Mix			Concrete
Statistical Parameter		Consistency mm	Air Content %	Density kg/m^3^	Compressive Strength MPa
Mean		128.0	1.70	2290	47.6
standard deviation		10.9	0.20	6.2	1.46
assigned value (robust mean)		130.0	1.80	2289	47.1
robust standard deviation		0.0	0.30	4.5	1.63
standard uncertainty of assigned value		0.0	0.12	1.8	0.68
expanded uncertainty of assigned value		0.0	0.28	4.2	1.5

**Table 8:** Correlation of *z-score* for the results of two methods: robust and classical statistics.

Property	Result Value/Method		Correlation of z-Score for the Results of Two Methods
	Classical Statistics: Mean Value; Standard Deviation	Robust Statistics: Robust Value; Robust Standard Deviation	
Consistency	127.78 mm	130.00 mm	0.991
	10.93 mm	0.00 mm	
Air content	1.71 %	1.80 %	0.814
	0.21 %	0.30 %	
Density	2289.92 kg/m^3^	2289.00 kg/m^3^	0.972
	6.22 kg/m^3^	4.45 kg/m^3^	
Compressive strength	47.58 MPa	47.10 MPa	0.906
	1.46 MPa	1.63 MPa	

## Data Availability

Data sharing not applicable.
